# STING-mediated type-I interferons contribute to the neuroinflammatory process and detrimental effects following traumatic brain injury

**DOI:** 10.1186/s12974-018-1354-7

**Published:** 2018-11-21

**Authors:** Amar Abdullah, Moses Zhang, Tony Frugier, Sammy Bedoui, Juliet M. Taylor, Peter J. Crack

**Affiliations:** 1 Neuropharmacology Laboratory, Department of Pharmacology & Therapeutics, University of Melbourne, Parkville, Melbourne, 3010 Australia; 2 Department of Microbiology & Immunology, Peter Doherty Institute, Melbourne, 3010 Australia

**Keywords:** STING, Type-I interferon, Traumatic brain injury, Neuroinflammation, Autophagy

## Abstract

**Background:**

Traumatic brain injury (TBI) represents a major cause of disability and death worldwide with sustained neuroinflammation and autophagy dysfunction contributing to the cellular damage. Stimulator of interferon genes (STING)-induced type-I interferon (IFN) signalling is known to be essential in mounting the innate immune response against infections and cell injury in the periphery, but its role in the CNS remains unclear. We previously identified the type-I IFN pathway as a key mediator of neuroinflammation and neuronal cell death in TBI. However, the modulation of the type-I IFN and neuroinflammatory responses by STING and its contribution to autophagy and neuronal cell death after TBI has not been explored.

**Methods:**

C57BL/6J wild-type (WT) and STING^−/−^ mice (8–10-week-old males) were subjected to controlled cortical impact (CCI) surgery and brains analysed by QPCR, Western blot and immunohistochemical analyses at 2 h or 24 h. STING expression was also analysed by QPCR in post-mortem human brain samples.

**Results:**

A significant upregulation in STING expression was identified in late trauma human brain samples that was confirmed in wild-type mice at 2 h and 24 h after CCI. This correlated with an elevated pro-inflammatory cytokine profile with increased TNF-α, IL-6, IL-1β and type-I IFN (IFN-α and IFN-β) levels. This expression was suppressed in the STING^−/−^ mice with a smaller lesion volume in the knockout animals at 24 h post CCI. Wild-type mice also displayed increased levels of autophagy markers, LC3-II, p62 and LAMP2 after TBI; however, STING^−/−^ mice showed reduced LAMP2 expression suggesting a role for STING in driving dysfunctional autophagy after TBI.

**Conclusion:**

Our data implicates a detrimental role for STING in mediating the TBI-induced neuroinflammatory response and autophagy dysfunction, potentially identifying a new therapeutic target for reducing cellular damage in TBI.

**Electronic supplementary material:**

The online version of this article (10.1186/s12974-018-1354-7) contains supplementary material, which is available to authorized users.

## Background

Traumatic brain injury (TBI) remains the leading cause of death and permanent disability in adolescents worldwide [1]. Current treatments are inadequate, with the majority of potential therapeutics failing in clinical trials [2–4]. This can be attributed to the complexities of the secondary damage and the pathways involved in the neuronal cell death after TBI that are not fully understood. TBI is characterised by an initial, irreversible damage at the site of impact with the brain, in severe cases, suffering extensive cell loss. This is followed by secondary injury leading to progressive neuronal cell death within the surrounding area [5] that is associated with priming of resident brain cells, mainly microglia and astrocytes, infiltration of peripheral leukocytes [6] and the subsequent release of inflammatory cytokines, chemokines [7] and other secondary messengers. Mounting evidence suggests neuroinflammation contributes to the neurological deficits observed after TBI [8–11]. It has been proposed that while this neuroinflammatory response contributes to a pro-survival milieu in the early stages of brain injury [12, 13], prolonged or chronic neuroinflammation is detrimental, leading to cell death in both animal studies and post mortem human brain samples [14–16]. Specifically, activation of microglia and astrocytes and the sustained release of pro-inflammatory cytokines such as TNF-α [17–19], IL-6 [20] and IL-1β [21–23] creates a toxic microenvironment detrimental to neuronal cell viability after brain injury.

The type-I interferons (IFNs) are known to be critical mediators of the inflammatory response in the periphery [24, 25]. Classically, the activation of type-I IFN signalling involves recognition of IFN-α and -β by their cognate receptors, composed of interferon receptor 1 (IFNAR1) and interferon receptor 2 (IFNAR2) subunits which are readily associated with the Janus activated kinases (JAKs) tyrosine kinase 2 (TYK2) and JAK1, respectively [26]. Upon activation, JAKs will in turn phosphorylate signal transducer and activator of transcription 2 (STAT2) or STAT1/3 at a tyrosine residue and subsequently activate the interferon regulatory factors (IRFs) (IRF3 and 7) [27, 28] leading to the production of type-I IFN and other pro-inflammatory cytokines [25]. Several recent studies have implicated the type-I IFNs in neuropathologies with increased expression linked to progression of neurological diseases including Gaucher disease [29], Aicardi-Goutieres syndrome [30] and model of prion disease [31]. In support of this, our group has identified a detrimental role for the type-I IFNs in animal models of Alzheimer’s (AD) [32, 33] and Parkinson’s disease (PD) [33] with elevated expression of type-I IFNs found in post-mortem human AD [33] and PD [34] brains. Furthermore, we also reported an increased expression of the type-I IFNs in human trauma brains (with greater than 6 h survival time) with attenuated type-I IFN signalling conferring a reduced neuroinflammatory response and smaller lesion volume in the controlled cortical impact (CCI) TBI animal model [35]. However, the underlying mechanisms that trigger the type-I IFN-mediated neuro-inflammatory response after TBI warrants further investigation.

The type-I IFNs can be alternatively activated through the stimulator of interferon genes (STING)-tumour necrosis factor (TNF) receptor-associated factor NF-κB activator (TANK)-binding kinase 1 (TBK1)-IRF3 signalling axis which requires the presence of cytosolic DNA in the cells. The STING-TBK1-IRF3 signalling pathway has long been appreciated as a trigger for DNA-dependent-IFN production. Aberrant IFN production signalling through a STING-dependent pathway has been implicated in autoinflammatory diseases [36] including vascular and pulmonary syndrome [37] and lupus [38]. STING has also been found to be upregulated in neurons infected by Japanese encephalitis viral RNA [39]. More recently, a study using cultured myeloid cells and mouse model of multiple sclerosis found that the antiviral drug ganciclovir (GCV) induces a type-I IFN response in microglia in a STING-dependent manner with activation of the STING pathway reducing microglial reactivity and the neuroinflammatory response [40]. Together, these studies suggest a role for STING in modulating immunological responses involving the type-I IFNs in the brain, with both neurotoxic and neuroprotective properties observed. However, the contribution of STING to the neuroinflammation occurring in acute and chronic neuropathologies is largely unknown.

Autophagy, a very well-characterised cellular degradation and/or recycling process, has also been implicated in human and animal models of TBI [41–44]. Following stimuli, autophagy is initiated by the formation of phagophore, which gradually elongates and envelops parts of cytoplasm such as damaged and old organelles. Eventually, propagating ends of the phagophore will come together to form a double-membrane vesicle termed the autophagosome, which subsequently fuse with the lysosome that degrades the materials captured by lysosomal hydrolases [45, 46]. Evidence in the literature reports increased autophagy markers after TBI, with both protective and detrimental effects observed. This double-edged sword role of autophagy reported after brain trauma may be due to the lack of understanding of its mechanisms and cell-type specificity within the CNS. Recently, a role for the STING and type-I IFN pathways in autophagy has also been proposed [47–50]. Specifically, STING and its downstream TBK1 protein are required for autophagy activation to eliminate bacterial infection in macrophages [51]. Subsequently, it was found that cyclic GMP-AMP synthase (cGAS), upstream activator of STING is required for activating type-I IFN production via the STING/TBK1/IRF3 pathway in this infection setting [52]. Intriguingly, cGAS is known to be degraded by p62-dependent selective autophagy upon sensing cytoplasmic DNA [53]. A recent report confirmed cGAS-STING degradation through this pathway is mediated by TBK1 [54] suggesting that the anti-microbial response and autophagy activation via STING is a tightly controlled event to prevent an excessive inflammatory response in the cells. However, the regulation of autophagy by STING and the type-I IFNs within the CNS is unknown. We were interested to investigate this in our CCI model and the possible role for STING and type-I IFN signalling in influencing this critical event after TBI.

In this study, we employed a similar CCI model as previously described by Karve et al. [35] to further elucidate the instigator of the type-I IFNs and its contribution to the neuroinflammatory environment after TBI. We hypothesise that type-I IFN expression is induced following TBI in part, through a DNA sensing pathway involving STING. Here, we report for the first time a critical role for STING in mediating the type-I IFN production and neuroinflammatory response after TBI. We found that STING^−/−^ mice subjected to CCI surgery have a smaller lesion size as compared to their wild-type (WT) littermates. Importantly, this neuroprotection can be attributed in part to reduced pro-inflammatory cytokine levels and reduced astrocyte activation. In addition, we observed increased STING mRNA levels in post mortem human TBI brains implicating a role for STING in acute brain injury. This study also provides the first evidence for a critical role for STING in modulating autophagy activity after TBI. STING^−/−^ mice had sustained and higher expression of autophagy markers including LC3 and p62 after TBI as compared to WT mice. Further, increased and impaired autophagic activity as measured by lysosomal-associated membrane protein 2 (LAMP2) expression levels was detected in WT mice at 24 h following CCI. However, reduced LAMP2 levels were identified in STING^−/−^ brains at 24 h post-TBI suggesting an adaptation to normal autophagic activity in the absence of STING after TBI. This increased autophagic activity might serve as protective mechanism to remove injured cells and promote a protective environment thus partially contributing to the neuroprotection observed in STING^−/−^ mice after TBI. Collectively, this study has identified a deleterious role for STING in mediating type-I IFN signalling and proposes STING as a potential target for therapeutic intervention following TBI.

## Methods

### Antibodies

Primary antibodies used for Western blot analysis:

**Table:** 

Primary antibodies	Origin	Dilution	Company	Catalogue no
Anti-TMEM173/STING	Rabbit	1 in 500	Abcam	ab92605
Anti-LC3	Rabbit	1 in 1000	MBL	PM036
Anti-SQSTM1/p62	Mouse	1 in 1000	Abcam	ab56416
Anti-GFAP(GA5)	Mouse	1 in 1000	Cell Signalling	#3670
Anti-β-Actin	Mouse	1 in 1000	Sigma-Aldrich	A5441
Anti-LAMP2	Rat	1 in 1000	Abcam	ab25339

Secondary antibodies used: horseradish peroxidize conjugated goat anti-rabbit (1: 1000, Dako, P0488), goat anti-mouse (1: 1000, Dako, P0447) and rabbit anti-rat (1:1000, Abcam, ab6734).

Primary antibodies used for immunohistochemical analysis:

**Table:** 

Primary antibodies	Origin	Dilution	Vendor	Catalogue no
Anti-TMEM173/STING	Rabbit	1 in 50	Abcam	ab92605
Anti-GFAP (GA5)	Mouse	1 in 1000	Cell Signalling	#3670
Anti-FOX3a	Mouse	1 in 250	Abcam	ab104224
Anti-IBA1	Rabbit	1 in 200	Wako	019–19741

Secondary antibodies used: Alexa fluor 488 goat anti-rabbit (1:1000, Life Technologies, A11008), Alexa fluor 594 goat anti-mouse (1:1000, Life Technologies, A11012).

### Animals

Adult male mice of 8–10 week of age with average body weight 23 ± 3 g were used in all experiments. WT mice of C57BL/6J background were purchased from the Animal Resource Centre while STING^−/−^ mice were a kind gift from Dr. Sammy Bedoui (Peter Doherty Institute, University of Melbourne) and Professor Ben Kile (Walter and Eliza Hall Institute, University of Melbourne).

### Controlled cortical impact

The controlled cortical impact (CCI) procedures performed in this study were based on standard protocols as previously described and reported by our group [11]. Briefly, mice were anaesthetised using ketamine (100 mg/kg, Parnell)/Xylazine (10 mg/kg, Parnell) via intra-peritoneal injection. Craniotomy was then performed with hand-held electrical drill (Dremel 10.8 V) removing the bone flap to expose the right parietal cortex. Mice were restrained by stereotaxic device and were subjected to a 1.5-mm deep impact (velocity of 5 m/s) using the computer-controlled impactor device (LinMot-Talk 1100) (impactor diameter of 2 mm). Sham control mice underwent identical procedures as those for CCI without actual injury by the impactor. Following successful CCI, all mice were euthanized at 2 h and 24 h post injury and brains removed for further analysis.

### Lesion size analysis

Twenty-four hours after CCI surgery, mice were transcardially perfused with 0.1% heparinised phosphate-buffered saline (Pfizer), followed by 4% paraformaldehyde (Scharlab S.L.) and their brains were isolated. Isolated brains were sectioned using a mouse brain matrix to 500 μm thickness followed by incubation in a 2% 2,3,5-triphenyltetrazolium chloride (TTC) in PBS solution at 35 °C for 15 min. Images of the stained brain sections were photomicrographed using a Zeiss Axioskop microscope and lesion area was determined using the ImageJ software (v1.47; NIH). White TTC staining of the lesion region within the brain was calculated using Cavalieri formula to find total lesion volume; [volume =  ΣA × *t* × ISF] where *A* =  sum of the corrected infarct areas, *t* =  section thickness (500 μm) and ISF =  inverse of the sampling fraction.

### RNA extractions and cDNA synthesis

Cortical and striatal regions of the brain were isolated from the ipsilateral and contralateral hemispheres and were homogenised in 1 ml Trizol (Invitrogen) before incubation at room temperature for 10 min. Then, 0.2 ml Chloroform (Chem Supply) per 1 ml Trizol was added to the samples, and samples were centrifuged at 12,000 g for 15 min at 4 °C to separate samples into phases. The colourless, aqueous phase of each sample, which contained RNA, was transferred into a fresh 1.7 ml microcentrifuge tube. RNA was precipitated by adding 0.5 ml Propan-2-ol (Chem Supply) per 1 ml Trizol, and samples were again centrifuged at 12,000 g for 10 min at 4 °C. The supernatant from the tubes was discarded, and the RNA pellet was washed with 75% Ethanol (Chem Supply) in diethyl pyrocarbonate (DEPC)-treated water (Sigma), vortexed and centrifuged at 7500 g for 5 min at 4 °C. The RNA pellet was air-dried, and redissolved in RNAse-free H_2_0 (Invitrogen). Concentration of the RNA samples was measured using the NanoDrop 1000 Spectrophotometer (Thermo Scientific).

### Quantitative real time polymerase chain reaction

cDNA was transcribed from 1 μg RNA using a high-capacity cDNA reverse transcription kit (Applied Biosystems) as previously described [33]. Genes of interest was detected using Taqman (Applied Biosciences) (Table 1) or SYBR green (GeneWorks) (Table 2) primers. Ct values were obtained for each sample, and relative transcript levels for each gene were calculated using the δδCT method [55]. For quantifying STING mRNA expression from human trauma samples, four control genes were used with the comparative C_T_ method (δC_T_) applied as previously described [11].

**Table 1:** Taqman primers used for QPCR analysis

Gene	Species	Refseq	Amplicon length (bp)	Catalogue no
GAPDH	Mouse	NM_008084.2	107	Mm99999915_m1
IFN-β	Mouse	NM_010510.1	69	Mm00439552_s1
IL1-β	Mouse	NM_008361.3	63	Mm01336189_m1
TNF-α	Mouse	NM_013693.3	81	Mm00443258_m1
IL6	Mouse	NM_031168.1	78	Mm00446190_m1
IRF3	Mouse	NM_016849.4	59	Mm00516779_m1
IRF7	Mouse	NM_001252600.1	67	Mm00516788_m1
		NM_001252601.1		
		NM_016850.3		
STING	Mouse	NM_028261.1	173	Mm01158117_m1
STING	Human	NM_001301738.1	89	Hs00736955_g1

**Table 2:** Sybr green primer sequences used QPCR analysis

Gene	Forward primer (5′-3′)	Reverse primer (5′-3′)
GAPDH	ATCTTCTTGTGCAGTGCCAGC	ACTCCACGACATACTCAGCACC
IFN-α	GCAATCCTCCTAGACTCACTTCTGCA	TATAGTTCCTCACAGCCAGCAG
IFNαE4	–	TATTTCTTCATAGCCAGCTG
TGF-β	TGCGCTTGCAGAGATTAAAA	CGTCAAAAGACAGCCACTCA

### QPCR analysis of human samples

Trauma brain samples from individuals who had died following closed head injury and non-head trauma controls were obtained from the Victorian Brain Bank Network (VBBN) [56] (Additional file 1: Table S1). RNA extractions and CDNA synthesis were performed as above with STING expression determined using Taqman primers (Table 1).

### Western blot analysis

Protein concentration was measured using Braford assay with 50 μg of protein used for Western blot analysis. Extracted proteins were incubated in 2× Novex® Tris-glycine SDS sample buffer (Invitrogen) for 10 min at 100 °C and were resolved on 8% or 12% acrylamide SDS PAGE gels. Blots were then transferred to polyvinylidene fluoride (PVDF) membranes using a semi-dry transfer apparatus (BioRad). Membranes were blocked with 5% *w*/*v* skim milk in TBS-T for 1 h and incubated with primary antibodies in 2% *w*/*v* skim milk in TBS-T at 4 °C overnight. Membranes were washed three times for 10 min each with TBS-T prior to being incubated with HRP-conjugated secondary antibodies (diluted in 2% skim milk in TBS-T) for 60 min at room temperature. Again, membranes were washed with TBS-T and signals were detected using an ECL prime® Western blotting detection kit (Amersham) and visualised with the IQ350 imaging machine (GE Healthcare). Post-image densitometry was performed using ImageJ software (NIH), whereby signal intensity was calculated in arbitrary units. For densitometry calculations, phosphorylation intensity was measured in arbitrary units and normalised to the β-actin loading control. These values were then calculated as fold change compared to control.

### Immunohistochemistry

Animals were perfused with ice-cold PBS followed by 4% PFA before hemispheres were removed and fixed in 5 ml of chilled 4% *w*/*v* paraformaldehyde, pH 7.4 (Sigma-Aldrich) overnight at 4 °C. These were then incubated at 4 °C overnight in 30% *w*/*v* sucrose before being embedded in OCT and cryosectioned into 30 μm coronal sections.

For immunohistochemistry, sections were permeabilised in 0.2% Triton X-100/PBS (PBS-T) for 20 min before being blocked for 1 h in 10% normal donkey serum/5% BSA/PBS at RT°C. The following antibodies were diluted in 1% BSA and incubated overnight at 4 °C: anti-mouse FOX3a (1:250, Abcam), anti-rabbit ionised calcium-binding adaptor molecule 1 (IBA1) (1:200, WAKO), anti-mouse glial fibrillary acidic protein (GFAP) (1:1000, Cell signalling) and anti-rabbit STING (1:50, Abcam). Sections were then washed three times in PBS before 2-h incubation at room temperature with Alexa Fluor 594-conjugated donkey anti-mouse and Alexa Fluor 488-conjugated donkey anti-rabbit secondary antibodies. Sections were again washed in three washes of PBS before being mounted in Vectashield plus DAPI (Vectashield). Slides were viewed using a Ziess Axio 123,672,641 microscope and images captured using an Axio Cam Mrm camera and Zen 2011 software. Three fields of view were taken of three sections/animal (Additional file 2: Figure S1).

### Statistical analysis

Data are expressed as mean ± SEM and were analysed using Graph Pad Prism 7.0 software. For QPCR data, a one-way analysis of variance (ANOVA) was performed followed by Bonferroni’s post-hoc analysis, with a value of *p* < 0.05 considered statistically significant. Lesion volume were analysed using an unpaired student’s *t* test, with a value of *p* < 0.05 considered statistically significant.

## Results

### STING expression is elevated in post-mortem human TBI brains

**Fig. 1:**
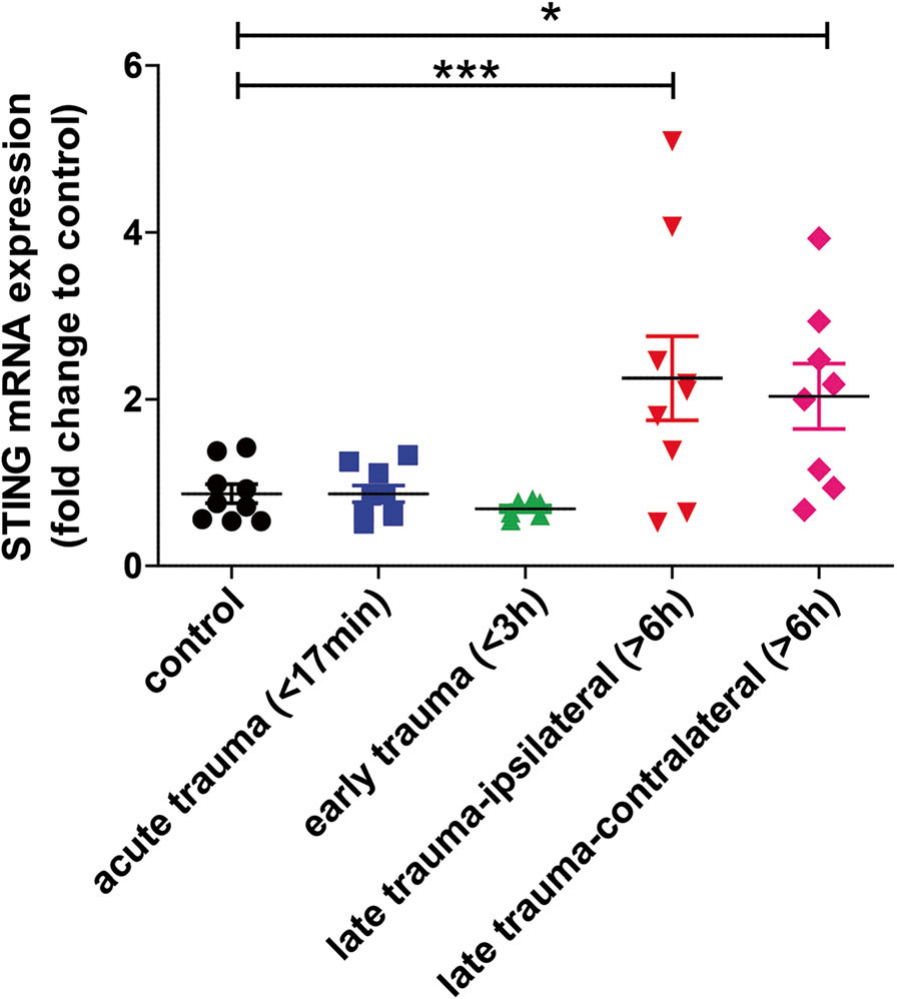
STING mRNA expression is upregulated in post-mortem human trauma brain samples. QPCR analysis identified increased mRNA expression of STING in late trauma group of post-mortem human trauma brains compared with controls (*n* = 8–10). Data represents mean ± SEM, *p < 0.05; ***p < 0.0001

To investigate a possible role for STING in TBI, mRNA expression was analysed by QPCR in post mortem human brain tissue. Details of post mortem human brain tissue can be found as previously described [35]. We found that STING mRNA level was significantly upregulated in both ipsilateral (2.729 ± 0.5082; ***p = 0.0003) and contralateral (2.193 ± 0.4101; *p = 0.0139) regions of late trauma group (patients died 6 h after TBI) as compared to control subjects (Fig. 1). This data implicates for the first time increased STING expression after TBI in human trauma brains.

### Increased levels of STING are detected following TBI

**Fig. 2:**
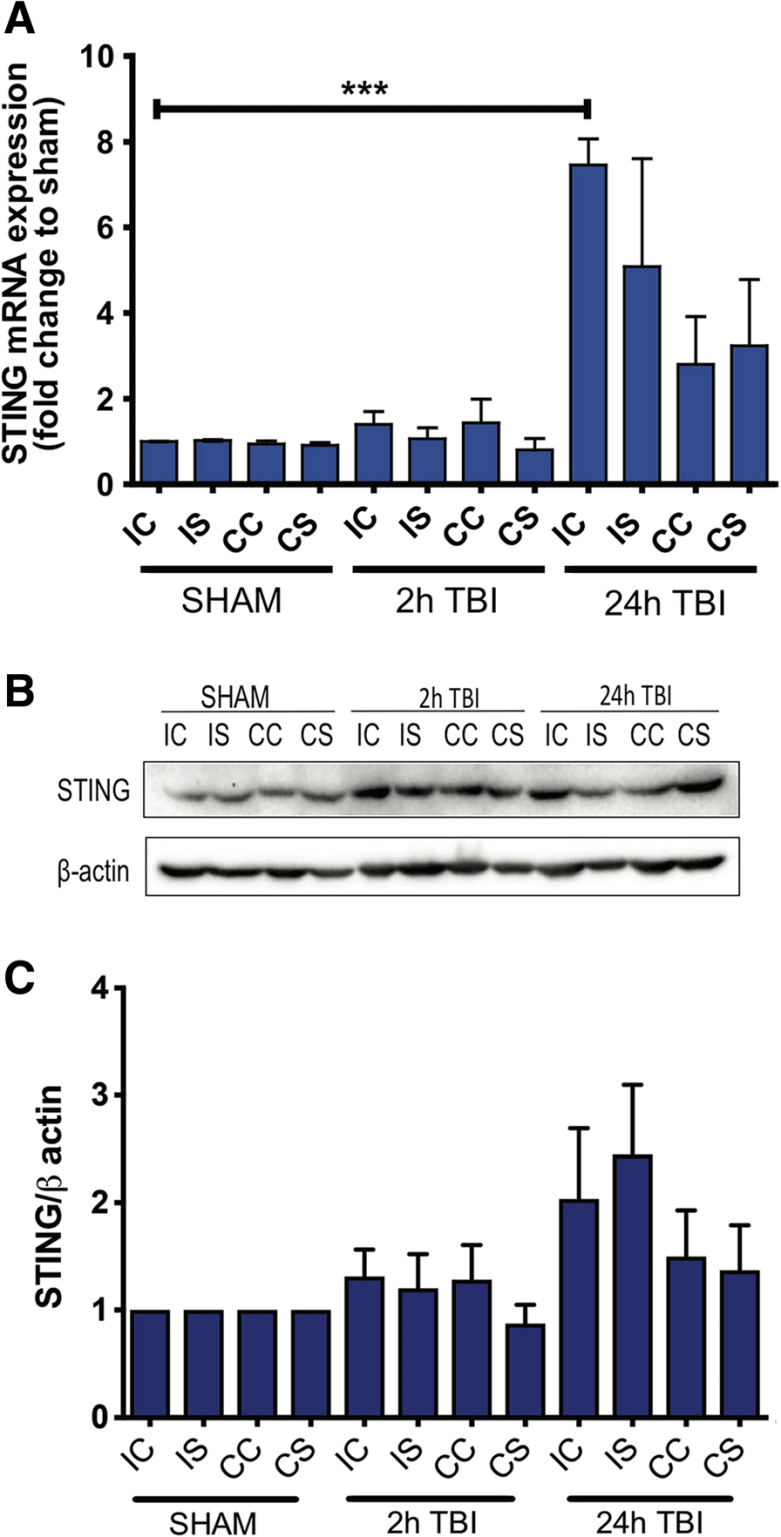
TBI induces STING expression in WT mice. Increased STING mRNA was detected by QPCR as shown in **a** (*n* = 6 for each time point) and Western blot analysis **b** (*n* = 3). Quantification of STING protein expression in (**b**) shown in (**c**). All data is expressed as mean ± SEM, ***p < 0.001. *IC* ipsilateral cortex, *IS* ipsilateral striatum, *CC* contralateral cortex, *CS* contralateral striatum

To further investigate the role of STING after TBI, WT mice were subjected to CCI surgery and brains removed at 2 h and 24 h after CCI for QPCR, Western blot and immunohistochemical analysis. Mice brains were divided into ipsilateral and contralateral regions, which were further divided into cortex and striatum. This allowed a critical assessment of the severity of the CCI model and its effects on gene expression in specific regions of the brain. STING mRNA expression was increased at 2 h with significant and robust upregulation at 24 h across ipsilateral and contralateral regions of the brain with the highest upregulation seen in the ipsilateral cortex (5.632 ± 0.8245; *p* = 0.0095) (Fig. 2a). This suggests that our CCI model induces global effects on the brain with the effect of the injury spreading throughout the brain. Elevated STING expression was confirmed at the protein level at 2 h and 24 h by Western blot analysis (Fig. 2b), although densitometric analysis found this to be not significant as compared to sham control; Sham IC VS 24 h TBI IC = 2.033 ± 0.6579; *p* = n.s. (Fig. 2c).

### STING colocalized with neuronal and astrocyte marker after TBI

**Fig. 3:**
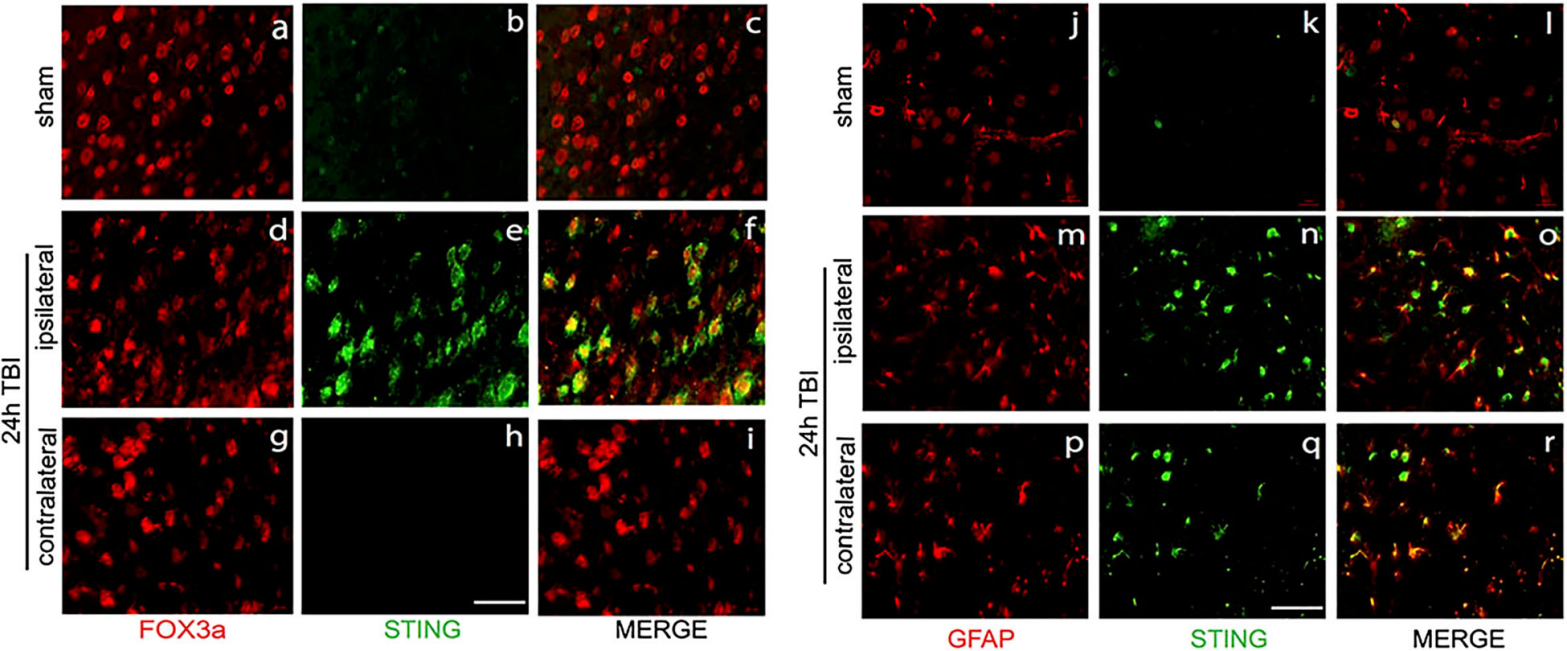
TBI induces STING expression in both neurons and astrocytes. Representative images of immunohistochemical analysis showing brain sections from the wild-type sham (**a**–**c** and **j**–**l**) and CCI (**d**–**i** and **m**–**r**) mice co-labelled with antibodies to identify neurons (FOX3a-positive cells; **a**–**i**) and astrocyte (GFAP-positive cell; **j**–**r**). Images are taken near lesion area (*n* = 3). Scale bar = 100 μM

To determine the cellular localisation of STING activation in the brain, we performed immunostaining on brain sections from WT mice after 24 h CCI. STING expression was detected near the lesion region and colocalized with FOX3a (neuronal marker) (Fig. 3f) with its expression barely detectable in the contralateral region (Fig. 3h) of the brain and sham sections (Fig. 3b). More interestingly, STING expression colocalized with GFAP (astrocyte marker) at 24 h after CCI in both the ipsilateral (Fig. 3o) and contralateral (Fig. 3r) regions. This suggests that astrocytic STING expression is widespread following TBI whilst neuronal expression of STING is restricted near the site of injury.

### STING^−/−^ mice exhibit a smaller lesion volume following TBI

**Fig. 4:**
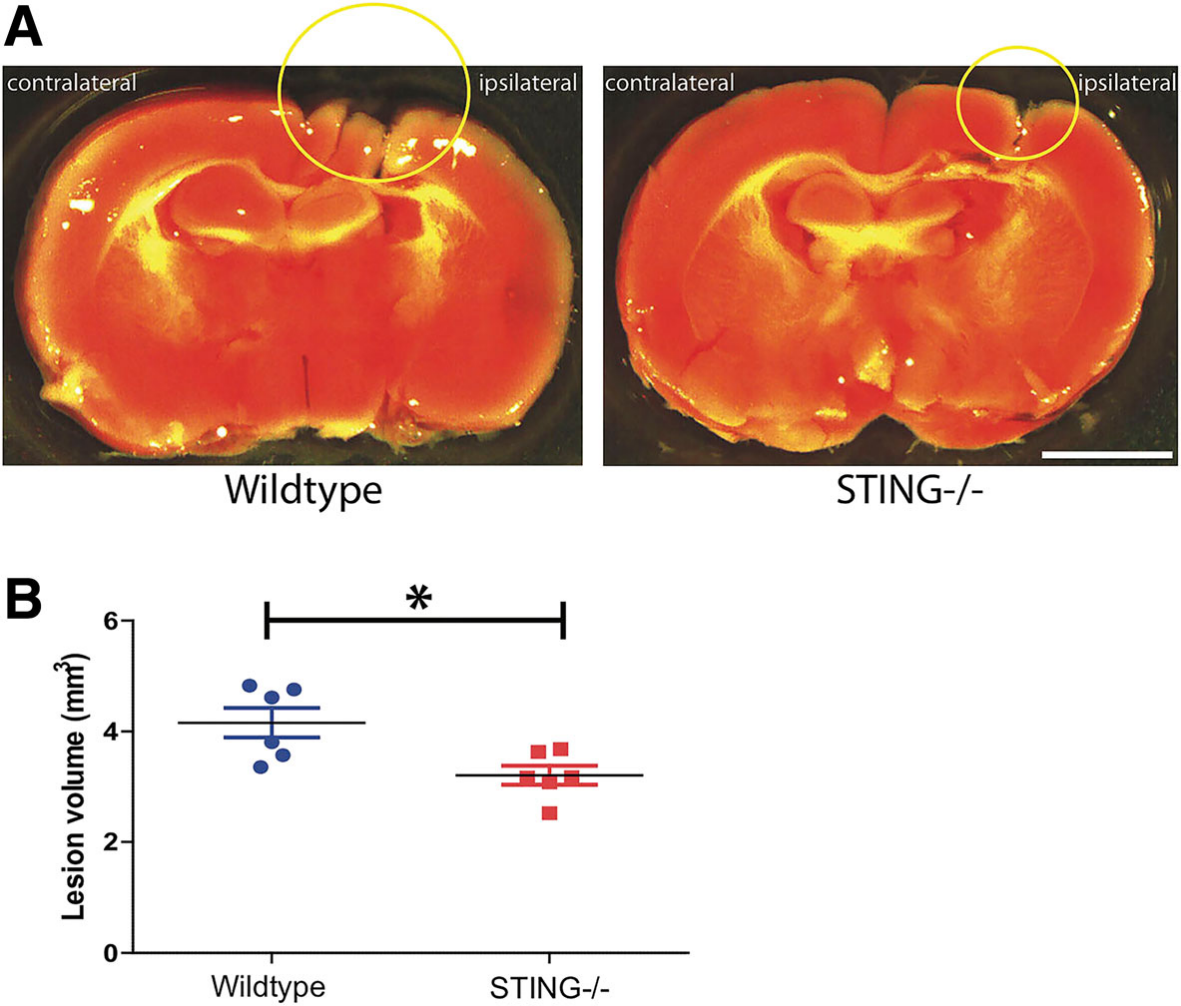
Genetic ablation of STING confers neuroprotection 24 h after TBI. Total lesion volumes of wild-type and STING^−/−^ mice were assessed by TTC staining and quantified using Image J. **a** Representative images demonstrating reduced infarct size in STING^−/−^ compared to wild-type mice 24 h after CCI (*n* = 6). **b** Quantification of (**a**) showing STING^−/−^ mice have significantly reduced lesion volumes compared to WT mice 24 h after CCI. Data represents mean ± SEM, *p* < *0.05; *n* = 6 animals per group. Scale bar: 1 mm

We recently reported that mice with reduced type-I IFN signalling (IFNAR1^−/−^ mice) displayed neuroprotection with reduced lesion size compared to their sham control group [35]. In this study, to further characterise the role of STING, TTC staining was performed on brain sections of WT and STING^−/−^ mice to measure the lesion size after CCI (Fig. 4a). STING^−/−^ mice had significantly smaller lesion size compared to their WT littermates (WT = 4.159 ± 0.2672 VS STING^−/−^ = 3.21 ± 0.1729; *p* = 0.0137) (Fig. 4b).

### TBI induces type-I IFN signalling in a STING-dependent manner

**Fig. 5:**
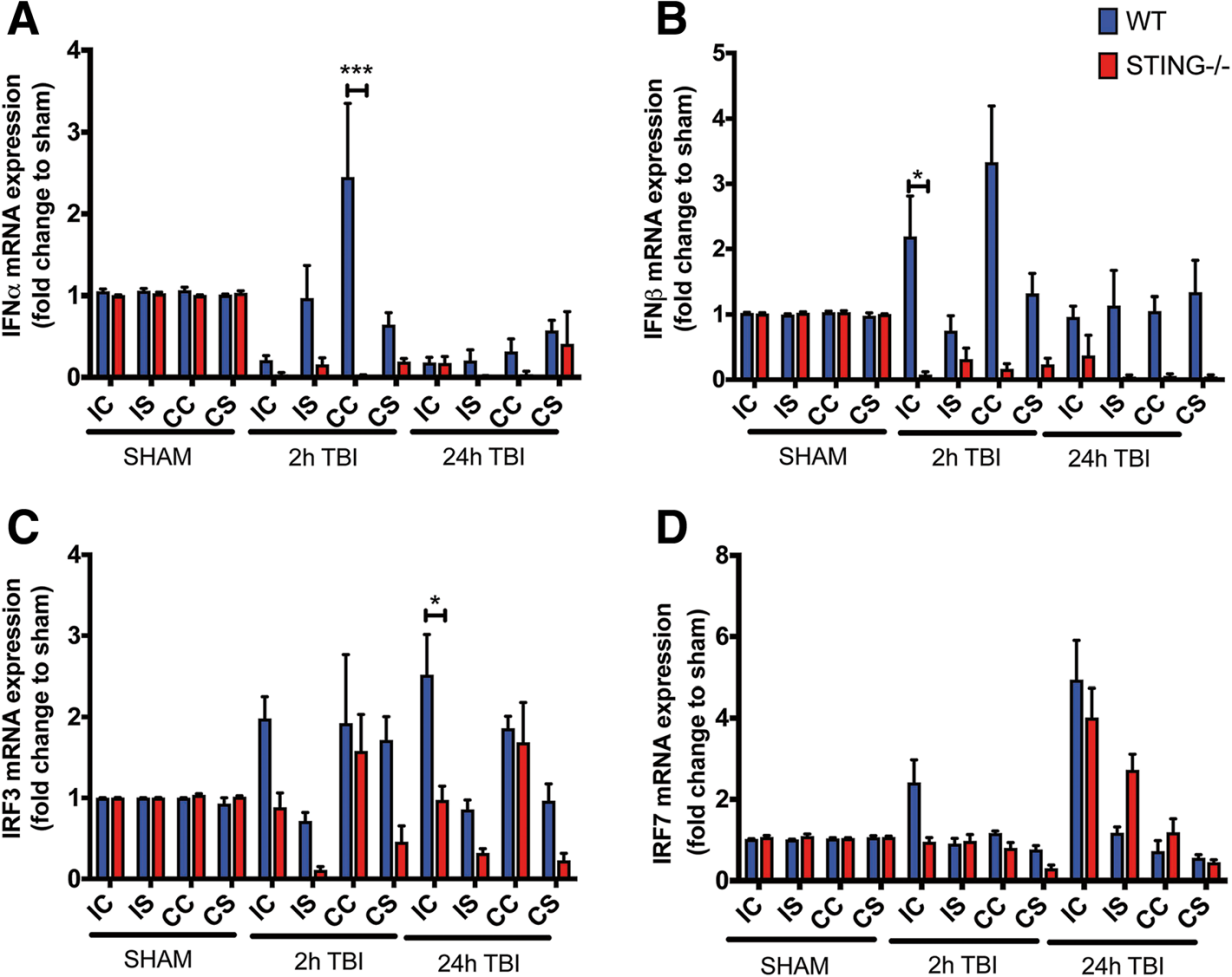
STING^−/−^ mice display reduced type-I IFN signalling after TBI. QPCR analysis identified increased mRNA expression of IRF3 (**a**), IRF7 (**b**), IFNα (**c**) and IFN-β (**d**) in WT brains mice as compared to STING^−/−^ mice after CCI (*n* = 6). Data represents mean ± SEM, *p < 0.05; ***p < 0.0001. *IC* ipsilateral cortex, *IS* ipsilateral striatum, *CC* contralateral cortex, *CS* contralateral striatum

To further characterise the STING pathway activation after CCI, we measured downstream STING effectors including IRF3 and IRF7 mRNA levels after 2 h and 24 h CCI (Table 3). Consistent with STING activation, we detected upregulation of IRF3 transcript levels at 2 h and 24 h TBI that was reduced in STING^−/−^ mice (Fig. 5a). Further, we also identified increased IRF7 mRNA levels in WT mice at 2 h and 24 h after CCI while STING^−/−^ mice showed an upregulation of IR7 at 24 h after CCI **(**Fig. 5b). These results suggest CCI induces IRF3 activation that is STING-dependent whilst IRF7 can be activated independently of STING at later time points after CCI. Next, to determine the role of STING in mediating the type-I IFN pathway after TBI, we analysed the type-I IFN expression profile (IFN-α and IFN-β) in WT and STING^−/−^ mice after CCI (Table 3). A significant and robust upregulation in IFN-β levels was detected in the ipsilateral cortex 2 h after CCI in the WT, but not STING^−/−^ mice (Fig. 5d). Twenty-four hours after CCI, we found that IFN-β expression returned to control levels in WT mice with STING^−/−^ mice showing reduced levels as compared to the control group. Similarly, increased expression of IFN-α was also detected in WT mice 2 h after CCI but reduced in STING^−/−^ mice as compared to the control group (Fig. 5c). Taken together, our data confirms the STING pathway is activated and supports our hypothesis that STING is an instigator of the type-IFN pathway after TBI.

**Table 3:** Neuroinflammatory gene expression changes in wild-type (WT) and STING^−/−^ mice at 2 h and 24 h post-TBI

	WT (2 h)	STING^−/−^ (2 h)	*p* value	WT (24 h)	STING^−/−^ (24 h)	*p* value
Expression cortex (relative to sham)						
IRF3	1.982 ± 0.269	0.883 ± 0.179	*p* = 0.7604	2.519 ± 0.496	0.973 ± 0.173	*p* = 0.0306
IRF7	2.417 ± 0.55	0.954 ± 0.104	*p* = 0.3106	4.938 ± 0.968	4.014 ± 0.721	*p* > 0.9999
IFN-α	2.450 ± 0.903	0.024 ± 0.007	*p* < 0.0001	0.180 ± 0.065	0.174 ± 0.082	*p >* 0.9999
IFN-β	2.191 ± 0.623	0.077 ± 0.044	*p* = 0.0492	0.963 ± 0.166	0.372 ± 0.3101	*p >* 0.9999
TNF-α	173.176 ± 23.243	1.170 ± 0.280	*p* < 0.0001	30.906 ± 14.556	0.136 ± 0.020	*p* = 0.6004
IL1-β	62.860 ± 18.641	2.340 ± 0.244	*p* < 0.0001	15.237 ± 5.301	3.290 ± 2.251	*p >* 0.9999
IL-6	6.322 ± 1.037	1.679 ± 0.489	*p* = 0.6419	10.198 ± 4.661	9.126 ± 2.532	*p >* 0.9999
Expression striatum (relative to sham)						
IRF3	0.715 ± 0.106	0.112 ± 0.039	*p >* 0.9999	0.856 ± 0.118	0.320 ± 0.053	*p >* 0.9999
IRF7	0.909 ± 0.130	0.971 ± 0.160	*p >* 0.9999	1.172 ± 0.148	2.727 ± 0.384	*p* = 0.1696
IFN-α	0.972 ± 0.394	0.159 ± 0.080	*p >* 0.9999	0.205 ± 0.133	0.012 ± 0.010	*p >* 0.9999
IFN-β	0.751 ± 0.231	0.315 ± 0.619	*p >* 0.9999	1.136 ± 0.534	0.045 ± 0.0268	*p >* 0.9999
TNF-α	138.223 ± 20.547	3.354 ± 0.868	***p < 0.0001	12.332 ± 4.273	0.388 ± 0.029	*p >* 0.9999
IL1-β	46.589 ± 10.443	6.324 ± 1.440	***p < 0.0001	3.260 ± 0.536	1.648 ± 0.362	n.s *p >* 0.9999
IL-6	8.086 ± 1.463	4.342 ± 0.690	*p* = 0.9244	0.484 ± 0.103	4.722 ± 0.884	*p* = 0.7951

### TBI-induced pro-inflammatory cytokines levels are reduced in STING^−/−^ mice

**Fig. 6:**
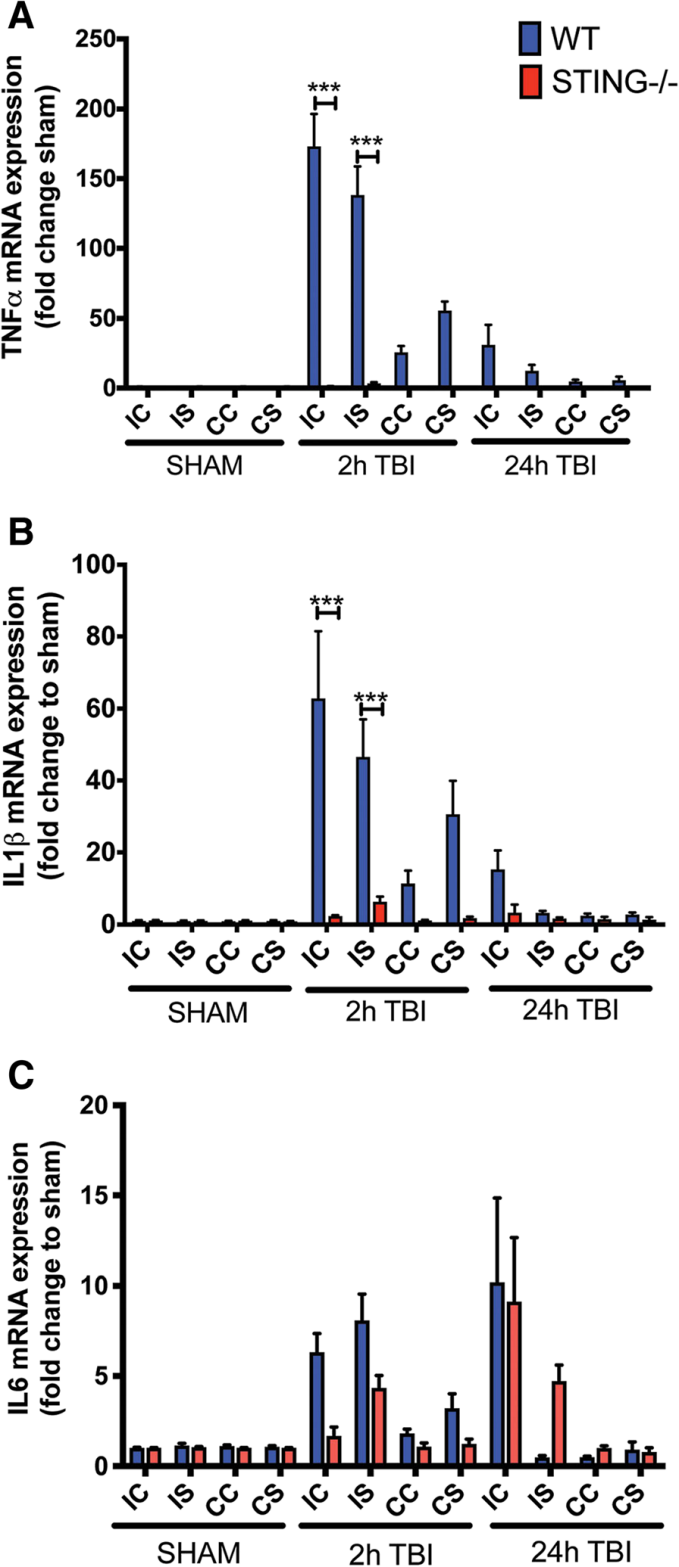
STING^−/−^ mice exhibit reduced TBI-induced pro-inflammatory cytokines in vivo. mRNA expression of IL-1β (**a**), IL-6 (**b**) and TNF-α (**c**) in brain tissue of wild-type and STING^−/−^ mice subjected to CCI. (*n* = 6 mice for each timepoint). Data represents mean ± SEM, *p < 0.05; **p < 0.01; ***p < 0.001. *IC* ipsilateral cortex, *IS* ipsilateral striatum, *CC* contralateral cortex, *CS* contralateral striatum

To elucidate the underlying mechanisms that contribute to the deleterious effect of STING after TBI, we determined the expression profile of the pro-inflammatory genes, TNF-α, IL-1β and IL-6 in WT and STING^−/−^ mice after CCI (Table 3). A significant and robust upregulation of TNF-α (Fig. 6a) and IL-1β (Fig. 6b) was detected in the ipsilateral side of WT mice with this diminished in STING^−/−^ mice at 2 h and 24 h after CCI. IL-6 levels were elevated in the ipsilateral cortex of WT mice 2 h after CCI compared to controls, while STING^−/−^ mice showed reduced expression at similar time point (Fig. 6c). However, 24 h after CCI, we observed an upregulation in IL-6 levels in the ipsilateral cortex both in WT and STING^−/−^ mice suggesting an alternative pathway that induces its expression independent of STING.

### STING contributes to astrocyte and microglia reactivity after TBI

**Fig. 7:**
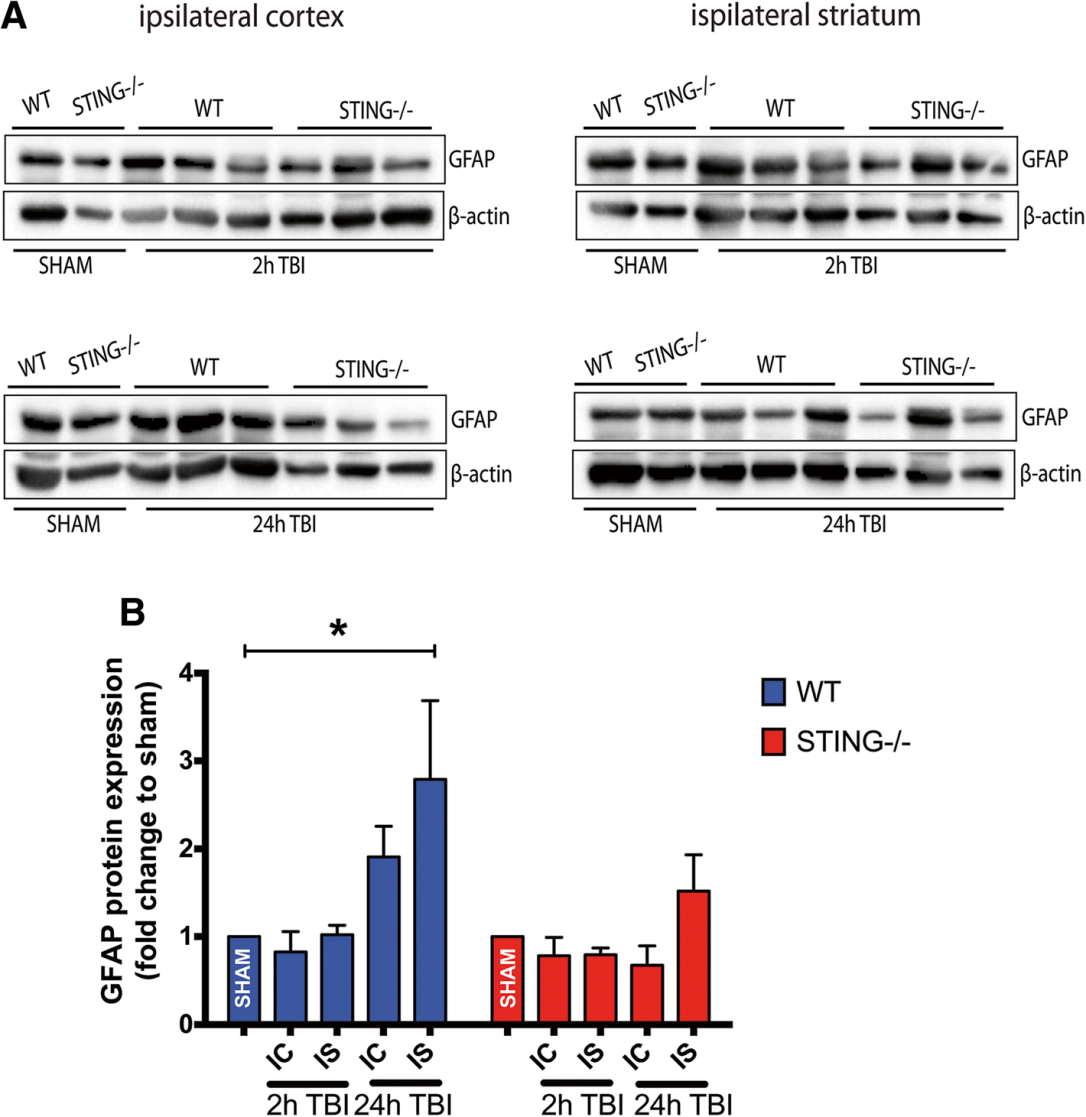
STING^−/−^ mice display reduced GFAP protein expression following TBI. **a** Representative images (*n* = 6 mice for each genotype and timepoint) showing GFAP protein levels in wild-type and STING^−/−^ mice after TBI as assessed by western blot analysis. Significantly increased expression of GFAP was observed in the ipsilateral striatum of wild-type mice 24 h post-TBI but not in STING^−/−^ mice as compared with sham group, as quantified in (**b**). Data represent mean ± SEM, *p < 0.05. *IC* ipsilateral cortex, *IS* ipsilateral striatum, *CC* contralateral cortex, *CS* contralateral striatum

**Fig. 8:**
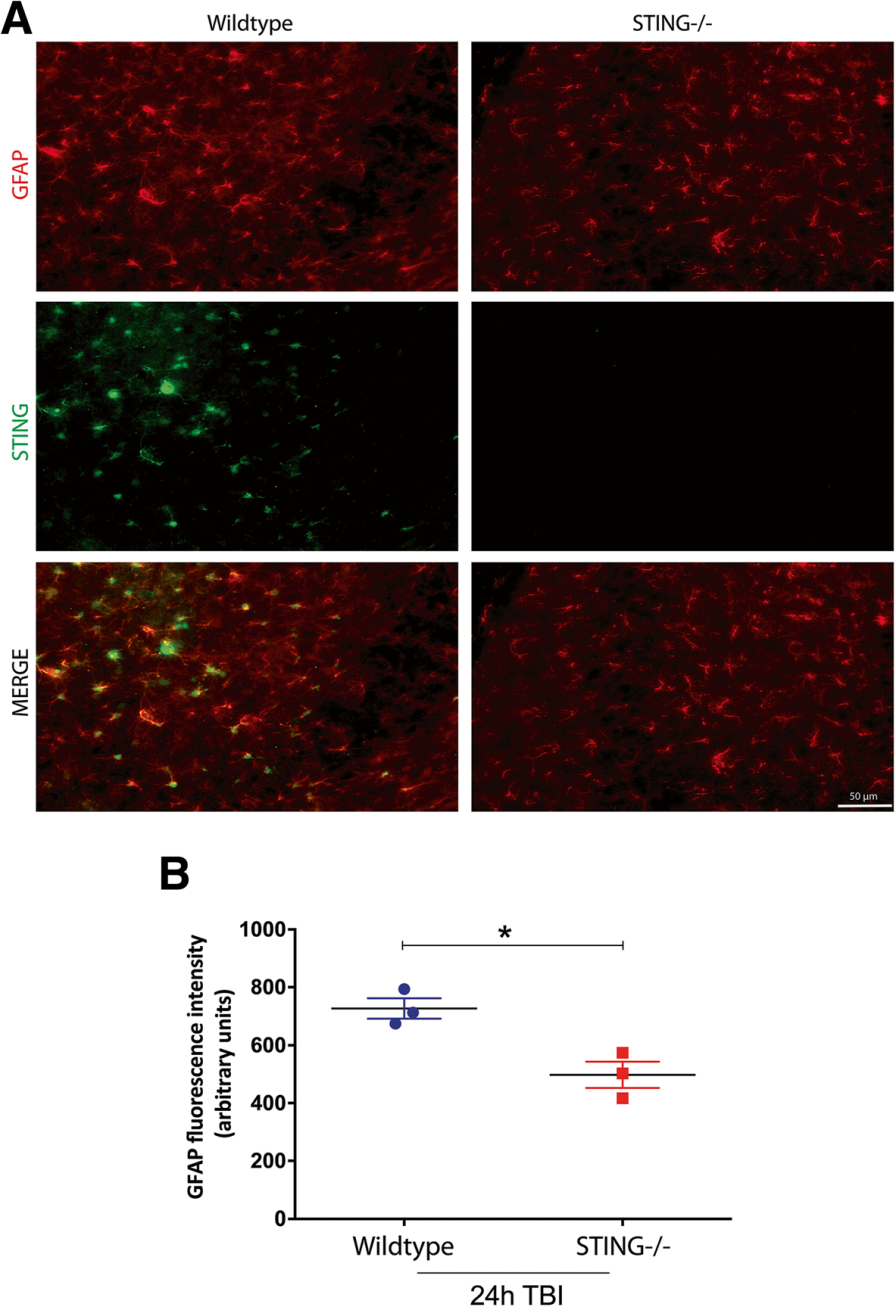
STING^−/−^ mice exhibit reduced GFAP immunostaining compared with WT mice after TBI. **a** High-power GFAP (red) staining co-labelled with STING (green) in the ipsilateral side of the brain. GFAP intensity is measured in (**b**) showing reduced GFAP immuno-reactivity in the STING^−/−^ mice 24 h after TBI (*n* = 3 mice for each genotype and timepoint). Data represent mean ± SEM, *p < 0.05

**Fig. 9:**
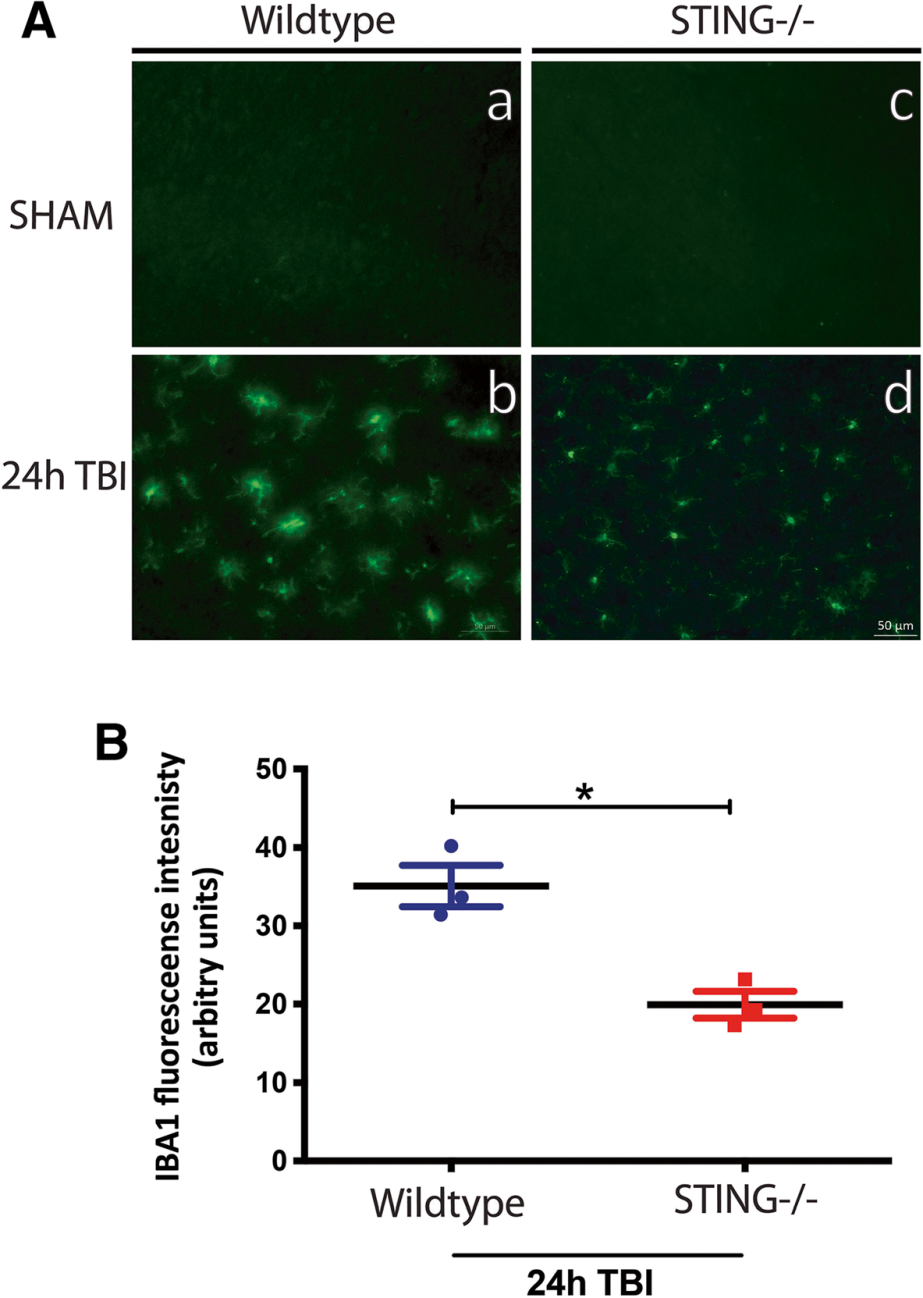
STING^−/−^ brains exhibit ramified microglial morphologies and reduced IBA-1 immunostaining following TBI. 24 h after TBI, brains from STING^−/−^ mice displayed microglia with ramified morphologies as identified by IBA-1 staining (*d*) as compared to WT mice (*b*). IBA-1 expression was significantly reduced in the STING^−/−^ mice at 24 h post-TBI as quantified in (**b**) (*n* = 3 mice for each genotype and timepoint)

To further understand the modulation of the neuroinflammatory response by STING after TBI, GFAP protein expression (an indicator of astrogliosis) was assessed in WT and STING^−/−^ mice 2 h and 24 h after CCI. An increase in GFAP protein expression was observed in WT TBI mice in the ipsilateral cortex and striatum 24 h after TBI (24 h IC = 1.909 ± 0.3485, 24 h IS = 2.788 ± 0.898; *p < 0.05 VS sham) with levels unchanged in STING^−/−^ (24 h IC = 0.6724 ± 0.222, 24 h IS = 1.519 ± 0.4144) mice as compared to sham as quantified by Western blot analysis (Fig. 7a, b). This was supported by immunohistochemical analysis with GFAP staining in WT and STING^−/−^ mice 24 h after CCI (WT = 726.4 ± 35.19 vs STING^−/−^ = 497.7 ± 45.5; *p = 0.0165) (Fig. 8c. Microglial activation is a common marker for neuroinflammation after CCI. We analysed IBA-1 immunofluorescence to determine a role for STING in modulating microglia activity after CCI in WT and STING^−/−^ mice. An activated form of microglia characterised by an amoeboid shape with a larger cell size was identified in the WT mice 24 h after CCI (Fig. 9b). In contrast, STING^−/−^ brains at 24 h post CCI displayed ramified morphologies with branches and fine processes indicative of a less reactive microglial phenotype (Fig. 9d). Further, 24 h after TBI the quantification of IBA-1 immunofluorescence intensity in STING^−/−^ mice showing significantly reduced staining as compared to wild-type brains (WT = 35.09 ± 2.637 vs STING^−/−^ 19.92 ± 1.709; **p = 0.0085). These results strongly support a role for STING in contributing to the detrimental neuroinflammatory environment by driving glial reactivity after CCI.

### STING contributes to autophagy dysfunction after TBI

**Fig. 10:**
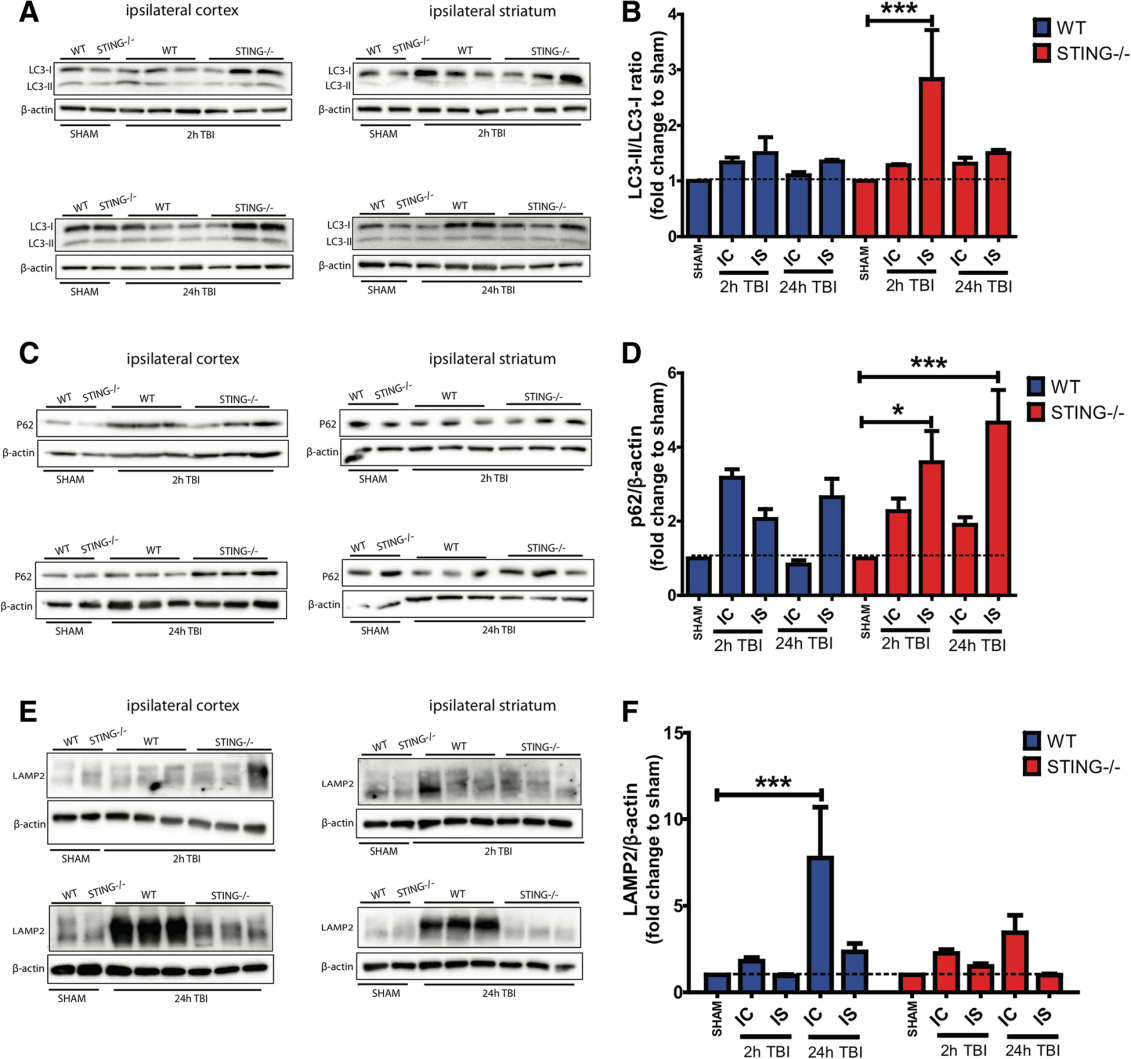
STING^−/−^ brains exhibit ramified microglial flux 24 h post-TBI. **a** LC3, (**c**) p62 and (**e**) LAMP2 expression was detected in wild-type and STING^−/−^ brains (*n* = 6) by Western blot. LC3-I, LC3-II, p62 and LAMP2 levels were normalised to β-actin levels respectively. For densitometry calculations, **b** LC3-II/LC3-I ratio, **d** p62/β-actin ratio and **f** LAMP2/β-actin ratio was then determined from these values and was calculated as a fold change relative to genotype sham control. Data is expressed as mean ± SEM, **p < 0.01; ***p < 0.001. *IC* ipsilateral cortex, *IS* ipsilateral striatum

STING has been implicated in influencing autophagy activity; therefore, we assessed its role in modulating autophagy after CCI. Hallmark autophagy markers, LC3, p62 and LAMP2, were measured 2 h and 24 h after CCI by Western blot analysis. An increase in LC3-II protein expression was observed in both WT and STING^−/−^ mice after CCI (Fig. 10b). Interestingly, our data showed a significant increase in LC3-II levels in the ipsilateral striatum 2 h after CCI in the STING^−/−^ mice as compared to the sham control but reduced in the WT mice (*LC3*-*II*/*LC3*-*I* ratio; WT 2 h IC = 1.598 ± 0.154 VS sham; n.s. *p* = 0.3509, WT 2 h IS = 1.877 ± 0.265 vs sham; *p = 0.0296, WT 24 h IC = 1.199 ± 0.080 vs sham; n.s. *p* = 0.9983, WT 24 h IS = 1.224 ± 0.085 vs sham; n.s. *p* = 0.9960, STING^−/−^ 2 h IC = 1.774 ± 0.209 VS sham; n.s. *p* = 0.0844, STING^−/−^ 2 h IS = 3.202 ± 0.372 vs sham; ***p < 0.0001, STING^−/−^ 24 h IC = 1.494 ± 0.116 vs sham; n.s. *p* = 0.6188, STING^−/−^ 24 h IS = 1.635 ± 0.095 vs sham; n.s. *p* = 0.2722). Further, elevated expression of p62 proteins levels was also detected in both WT and STING^−/−^ mice after CCI with STING^−/−^ mice showing higher expression at 2 h and 24 h as compared to control genotypes (Fig. 10d) (*p62*/*β*-*actin*; WT 2 h IC = 3.175 ± 0.145 vs sham; ***p < 0.0001, WT 2 h IS = 2.052 ± 0.209 VS sham; n.s. *p* = 0.1292, WT 24 h IC = 0.931 ± 0.104 vs sham; n.s. *p* > 0.9999, WT 24 h IS = 2.422 ± 0.281 vs sham; **p = 0.0088, STING^−/−^ 2 h IC = 2.300 ± 0.206 vs sham; *p = 0.0231, STING^−/−^ 2 h IS = 3.783 ± 0.427 VS sham; ***p < 0.0001, STING^−/−^ 24 h IC = 1.8688 ± 0.192 vs sham; n.s. *p* = 0.3461, STING^−/−^ 24 h IS = 4.572 ± 0.486 vs sham; ***p < 0.0001). To determine whether the increases in LC3-II and P62 levels were due to an increase in autophagy activity or impaired autophagy flux as a result of a block in the autophagosome-lysosomal degradation step, we examined LAMP2 levels. As expected, WT mice showed a significant increase in LAMP2 levels in the ipsilateral cortex 24 h after CCI as compared to sham control (WT 24 h IC = 7.761 ± 2.927 vs sham; ***p < 0.0001). However, reduced LAMP2 levels were detected in the STING^−/−^ mice at 24 h after CCI as compared to the control and WT genotypes (STING^−/−^ 24 h IC = 1.440 ± 0.190 vs sham; n.s. *p >* 0.9999) (Fig. 10e). It is noteworthy that LC3 and p62 levels were also higher as compared to LAMP2 levels in the STING^−/−^ mice subjected to CCI, suggesting enhanced normal autophagy activity with complete autophagosome-lysosomal degradation. This increased autophagic flux could contribute to the neuroprotective effects observed in the STING^−/−^ mice after CCI.

## Discussion

Neuroinflammation is known to be a key driver of secondary injury progression after TBI; however, its precise mechanisms remain unclear. Identifying key molecules that regulate neuroinflammatory processes could lead to a potential therapeutic target to improve patients’ outcome following TBI. This study stems from our previous finding that type-I IFNs contribute to the detrimental neuroinflammatory environment in a CCI animal model of TBI. Mice lacking the type-I IFN receptor (IFNAR1^−/−^) or by targeting the IFNAR1 receptor with a blocking monoclonal antibody conferred protection after TBI [35]. Here, we sought to determine the instigator of this type-I IFN production that leads to an increased pro-inflammatory environment in this CCI model. For the first time, we report a novel role for STING in mediating neuroinflammatory processes after TBI. Genetic ablation of STING leads to decreased type-I IFN production, a reduction in pro-inflammatory cytokine expression including TNF-α, IL-6 and IL-Iβ and significantly a reduced lesion volume 24 h post CCI. In addition, this study highlights for the first time a role for STING in regulating autophagy activity with STING^−/−^ mice displaying increased autophagic flux in their brains after CCI.

We have previously demonstrated a detrimental role for the type-I IFNs in acute and chronic neurodegenerative diseases with increased type-I IFN expression found in both animal models and post-mortem human brains from TBI [35], AD [33] and PD [34] patients. In these animal models, reduced type-I IFN signalling was associated with an attenuated neuroinflammatory response and subsequent neuroprotection. The underlying mechanisms mediating the detrimental effects of the type-I IFNs are still not well understood. This study aimed to further characterise the signalling pathways that contributed to the increased type-I IFN production, specifically in the CCI model. Type-I IFNs can signal through the classical JAK/STAT-IRF7 pathway leading to an upregulation in pro-inflammatory cytokines and release of the type-IFN themselves [57]. The released type-I IFNs can further bind to the IFNAR1 receptor in a positive-feedback mechanism thus enhancing this signalling [58]. Alternatively, type-I IFNs can be activated through cytosolic DNA via the STING-TBK1-IRF3 pathway. The STING-dependent type-I IFNs signalling has been very well characterised in infectious disease settings; however, its role in neuroinflammation is unclear. Here, for the first time, we confirmed increased STING mRNA levels in post-mortem human TBI brains. Increased STING expression was detected in the late trauma group (patients who died > 6 h after TBI) in both the ipsilateral and contralateral sides as compared with the control group. Interestingly, IFN-β was only increased in the ipsilateral side (not in the contralateral side) of the late trauma group while IFN-α was significantly reduced in the early trauma group (patients who died < 3 h after TBI) as previously reported [35]. This implies a co-activation of the STING and type-I IFN pathways after TBI and suggests a dominant IFN-β production after injury. The increased STING expression in both the contra- and ipsilateral sides of human TBI brains in the late trauma group implicates STING in mediating the progression of the neural injury.

Consistent with our findings in human TBI samples, we confirmed increased STING mRNA expression at 2 h after CCI with a robust and a significant upregulation at 24 h in the ipsilateral cortex as compared to the sham control. STING mRNA expression was also higher across both the ipsilateral and contralateral sides at 24 h after TBI as compared to the control group with Western blot confirming this at the protein level. In addition, we confirmed downstream STING and type-I IFN signalling activation in our CCI model with increased IRF3 and IRF7 mRNA levels detected in WT mice at 2 h and 24 h after TBI. However, STING^−/−^ mice showed reduced IRF3 expression in the ipsilateral cortex at 2 h and 24 h after TBI as compared to the WT genotypes. Further, increased IRF7 mRNA was only detected at 24 h after TBI in the STING^−/−^ mice as compared to the WT brains suggesting that there is alternative pathway mediating IRF7 activation at later timepoints in the absence of STING. These results suggest that CCI induces both IRF3 and IRF7 production and is partially STING-mediated. Numerous reports have confirmed that STING can be activated by cyclic dinucleotides and/or double-stranded (ds) DNA produced by bacteria or virally transfected cells [59–61]. STING has also been shown to be upregulated by self-DNA released during cell death in animal models of liver disease [62–64] and UV-irradiation-induced cell death [65]. More recently, activation of the STING-IRF3 axis has been implicated in driving the inflammatory response and apoptosis in fatty liver disease [66]. As CCI induces injury and tissue damage in the proximity of cortical regions of the brain, it can be implied that STING expression is induced by the release of self-DNA from the injured or dying cells after CCI. It is also possible that other type of damage-associated molecule patterns (DAMPs) might activate STING after TBI. The exact molecules or mechanism of action activating STING in our CCI model warrants further investigation. In addition, it would be of interest to further investigate the long-term contribution of STING by extending the period after injury up to 7 days or longer and/or increasing the depth and velocity of impact which correlates with severity of cortical deformation [41, 67–69].

Microglial and astrocyte reactivity are common neuroinflammatory features after TBI. We found that STING contributes to the increased IBA1 staining and altered microglial morphology after CCI. We also observed increased GFAP expression at 24 h after TBI in WT mice but significantly reduced in STING^−/−^ mice at similar time points. This reduction in STING-dependent glial reactivity after TBI may contribute to the neuroprotective effects seen in STING^−/−^ mice. Recently, it was reported that STING activation reduces microglial reactivity in a multiple sclerosis (MS) animal model [40]. This dual function of STING in regulating microglial reactivity observed may be attributed to the different disease models; however, it does suggest an important role for STING in contributing to the neuroinflammatory response in these CNS pathologies. We confirmed STING expression near the lesion site colocalized with GFAP (astrocyte) and FOX3a (neurons) positive cells. However, only GFAP-positive cells coexpressing STING were found in both the ipsilateral and contralateral sides 24 h after TBI suggesting that astrocytes are the major cell involved in the STING-mediated response after TBI. Utilising a bone marrow chimera approach, we have previously confirmed that type-I IFNs produced by the peripheral tissue compartment are a major contributor to the pro-inflammatory response after TBI [35]. The possibility that the TBI-induced activation of the STING pathway is systemic or brain-derived in origin warrants further investigation; however, we did confirm a critical role for STING in mediating the neuronal cell death in our CCI model. STING^−/−^ mice exhibited a reduced lesion volume compared to their wild-type littermates consistent with our observation with IFNAR1^−/−^ mice which display a significantly smaller lesion area compared to sham controls [35]. This implies that both STING and type-I IFN signalling are crucial players in exacerbating the outcome of TBI. TBI is known to induce a pro-inflammatory environment with a prolonged or chronic response exacerbating TBI outcome. IFN-β mRNA levels were upregulated at 2 h after TBI in WT mice but not in STING^−/−^ mice, suggesting that the increased type-I IFN expression is through a STING-dependent pathway. We also found a dramatic reduction in mRNA expression of TNF-α and IL-1β in STING^−/−^ mice as compared to their WT littermates at 2 h and 24 h after TBI. Whilst it is known that STING modulates pro-inflammatory cytokine production in a bacterial or viral infection setting [70, 71], its role in CNS injury is unknown. Based on our findings, we conclude that the attenuated pro-inflammatory cytokine and IFN-β levels contribute to the neuroprotective effects observed in STING^−/−^ mice.

The role of autophagy has been widely implicated in TBI with increased autophagic marker expression observed in both human and animal models [41, 44, 72]. However, its precise role and the mechanisms that trigger its induction after TBI remain unclear. Both the STING and type-I IFN pathways have emerged as key players in autophagy activation in cellular and other disease models [47, 51] but their interaction and regulation following brain injury is unknown. Our results demonstrated increased expression of autophagy markers in both WT and STING^−/−^ mice at 2 h and 24 h after TBI as compared to their sham controls validating our CCI model in inducing autophagy as previously reported [44, 73, 74]. Interestingly, after CCI, we observed increased and sustained expression of LC3 and p62 in STING^−/−^ brains as compared to their WT counterparts. Autophagy is a dynamic and complex cellular degradation process that requires a careful analysis to accurately identify and interpret its activity to understand if dysfunction is occurring due to increased initiation or decreased flux. In this study, we assessed hallmark markers of autophagy including microtubule-associated protein 1 light chain 3 (LC3), SQSTM1/p62, and lysosomal-associated membrane protein 2 (LAMP2) in our CCI model. Conversion of LC3-I to LC3-II is representative of increased autophagy activation [75] while the degradation of p62 and LAMP2 at the late step of the autophagy process serves as a marker for normal autophagic flux. The increased expression of LC3-II and p62 levels at 2 h and 24 h time points in the WT mice after CCI in this study corresponds to other studies that detected increased expression of these autophagic markers up to 7 days after TBI [44]. Interestingly, our data showed higher and sustained expression of LC3-II and p62 levels in STING^−/−^ mice at 2 h and 24 h after TBI as compared to their WT counterparts. Given that we observed a neuroprotective effect in STING^−/−^ at 24 h after TBI, this suggests that the increased LC3-II and p62 levels observed are not an indication of impaired autophagy flux but rather enhanced autophagy activity that serves as a protective mechanism to reduce cellular damage following TBI. Indeed, we confirmed decreased expression of LAMP2 levels at 2 h and 24 h in the ipsilateral cortex after CCI in STING^−/−^ mice as compared to their WT counterparts. This reduced LAMP2 expression in the STING^−/−^ mice indicates that there is a completion of the autophagy process suggesting that STING might be a key regulator driving autophagic dysfunction seen after TBI. The dynamic process of autophagy and a clear understanding of STING function in regulating this event after TBI can be tackled by incorporating longer time points after CCI which are lacking in the current study.

## Conclusions

Taken together, this study provides evidence to suggest a novel role for STING in mediating the type-I IFN pathway in the CCI model of TBI. This is also the first study to demonstrate the influence of STING in regulating the autophagy pathway following TBI. Our finding that STING is a key regulatory protein involved in TBI has identified a novel potential therapeutic strategy for the reducing the damage following brain injury.

## Additional files

Additional file 1: **Table S1.** Details of trauma and non-trauma control cases. (DOCX 20 kb)

Additional file 2: **Figure S1.** Representative diagrams demonstrating brain regions (marked by black box) assessed for Iba-1, GFAP and Fox3a immunostaining. (JPG 1061 kb)

## Abbreviations

AD: Alzheimer’s disease
CCI: Controlled cortical impact
GFAP: Glial fibrillary acidic protein
IBA1: Ionised calcium-binding adaptor molecule 1
IFN: Interferon
IFNAR1: Interferon alpha receptor 1
IFNAR2: Interferon alpha receptor 2
IL: Interleukin
IRF: Interferon regulatory factor
JAK: Janus activated kinase
LAMP2: Lysosomal-associated membrane protein 2
LC3: Including microtubule-associated protein 1 light chain 3
PD: Parkinson’s disease
STAT: Signal transducer and activator of transcription
STING: Stimulator of interferon genes
TBI: Traumatic brain injury
TBK: Tumour necrosis factor (TNF) receptor-associated factor NF-κB activator (TANK)-binding kinase 1 (TBK1)
TNF: Tumour necrosis factor
TYK2: Tyrosine kinase 2
