# Supplementary material for: STING-mediated type-I interferons contribute to the neuroinflammatory process and detrimental effects following traumatic brain injury
Source: J Neuroinflammation. 2018 Nov 21;15:323. doi: 10.1186/s12974-018-1354-7 (PMC6247615; doi:10.1186/s12974-018-1354-7)
Supplement: Supplementary file 1 — Table S1. Details of trauma and non-trauma control cases. (DOCX 20 kb) [file 12974_2018_1354_MOESM1_ESM.docx]

**Table S1: Details of trauma and non-trauma control cases.**

| **TABLE:** Details of 27 trauma and 10 control cases | | | | | | |
| --- | --- | --- | --- | --- | --- | --- |
| **Case** | **Age** | **Sex** | **Cause of Injury** | **PMI, h** | **Cause of death** | **Survival time** |
| 1 | 51 | M | Motor vehicle accident | 60 | Brain + multiple injuries | < 17 mins |
| 2 | 63 | M | Household accident | 70 | Brain injury | < 17 mins |
| 3 | 27 | M | Suicide | 84 | Brain + multiple injuries | < 17 mins |
| 4 | 41 | M | Suicide | 96 | Brain + multiple injuries | < 17 mins |
| 5 | 57 | F | Motor vehicle accident | 87 | Brain + multiple injuries | < 17 mins |
| 6 | 49 | M | Motor vehicle accident | 107 | Brain + multiple injuries | < 17 mins |
| 7 | 45 | M | Motor vehicle accident | 43 | Brain + multiple injuries | < 17 mins |
| 8 | 21 | M | Motor vehicle accident | 100 | Brain injury | < 17 mins |
| 9 | 41.3 | M | Aviation accident | 114 | Brain + multiple injuries | < 17 mins |
| 10 | 57.6 | F | Motor vehicle accident | 97 | Brain injury | < 17 mins |
| 11 | 16.8 | M | Motor vehicle accident | 85 | Brain + multiple injuries | < 3 hrs |
| 12 | 78.7 | M | Household accident | 45 | Brain injury | < 3 hrs |
| 13 | 18.3 | M | Motor vehicle accident | 79 | Brain + multiple injuries | < 3 hrs |
| 14 | 34.7 | M | Motorbike accident | 66 | Brain + multiple injuries | < 3 hrs |
| 15 | 22.9 | F | Motor vehicle accident | 108 | Brain + multiple injuries | < 3 hrs |
| 16 | 52.8 | M | Motorbike accident | 65 | Brain + multiple injuries | < 3 hrs |
| 17 | 19.6 | M | Suicide | 33 | Brain + multiple injuries | < 3 hrs |
| 18 | 59.8 | M | Motor vehicle accident | 71 | Brain + multiple injuries | < 3 hrs |
| 19 | 46.0 | M | Fall | 129 | Brain injury | 6 hrs |
| 20 | 56.3 | M | Motor vehicle accident | 65 | Brain injury | 8 hrs |
| 21 | 64.6 | M | Fall | 61 | Brain injury | 8 hrs |
| 22 | 75.9 | M | Staircase fall | 89 | Brain injury | 10 hrs |
| 23 | 59.6 | F | Motor vehicle accident | 80 | Brain injury | 35 hrs |
| 24 | 61.7 | M | Fall | 40 | Brain injury | 93 hrs |
| 25 | 38.9 | F | Staircase fall | 101 | Brain injury | 122 hrs |
| 26 | 70.9 | M | Motor vehicle accident | 114 | Brain injury | 76 hrs |
| 27 | 73.7 | M | Fall | 91 | Brain injury | 29 hrs |
| Controls |  |  |  |  |  |  |
| 28 | 16 | M | - | - | Suicide by hanging | - |
| 29 | 48.7 | M | - | 50 | Cardiac failure | - |
| 30 | 51.6 | M | - | 64 | Asthma | - |
| 31 | 52.3 | M | - | 52 | Cardiomyopathy | - |
| 32 | 59.6 | M | - | 43 | Pulmonary embolism | - |
| 33 | 64.1 | M | - | 24 | Ischaemic heart disease | - |
| 34 | 66.9 | M | - | 10 | Pneumonia | - |
| 35 | 64.4 | M | - | 24 | Pulmonary embolism | - |
| 36 | 77.5 | M | - | 53 | Myocardial infarction | - |
| **37** | 60 | F | - | 48 | Myocardial infarction | - |
| Cases 1-10: cases with a survival time between 0 and 17 minutes; Cases 11-18: cases with a survival time between 30 minutes and 3 hours; Cases 19-27: cases with a survival time between 6 and 261 hours; Cases 28-37: control cases. All brains were obtained at autopsy. PMI, *post mortem* interval (time between death and brain retrieval); M, male; F, female. | | | | | | |
