# Supplementary figures and images for: STING-mediated type-I interferons contribute to the neuroinflammatory process and detrimental effects following traumatic brain injury
Source: J Neuroinflammation. 2018 Nov 21;15:323. doi: 10.1186/s12974-018-1354-7 (PMC6247615; doi:10.1186/s12974-018-1354-7)

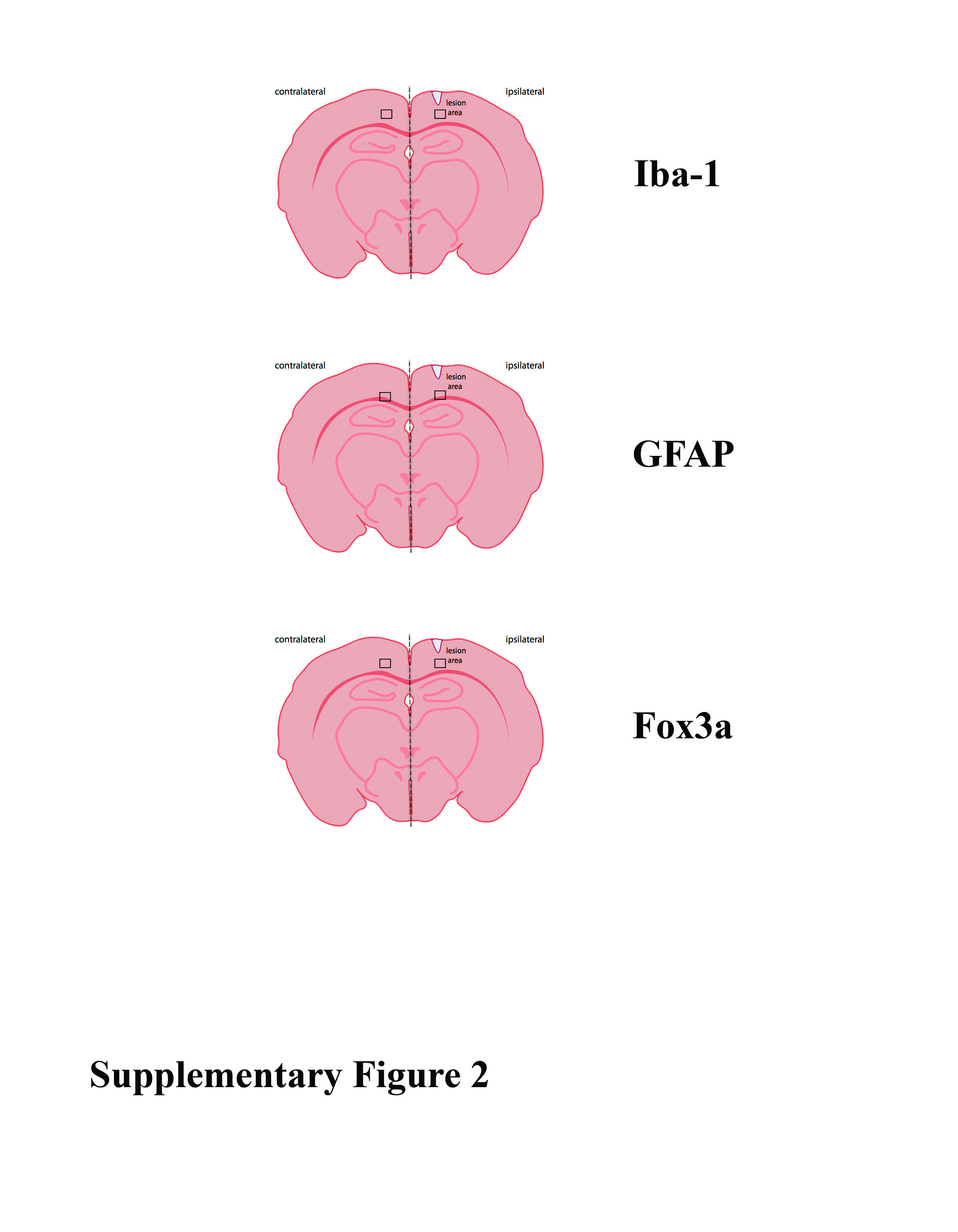

Supplement: Supplementary file 2 — Figure S1. Representative diagrams demonstrating brain regions (marked by black box) assessed for Iba-1, GFAP and Fox3a immunostaining. (JPG 1061 kb) [file 12974_2018_1354_MOESM2_ESM.jpg]
